# NPEBseq: nonparametric empirical bayesian-based procedure for differential expression analysis of RNA-seq data

**DOI:** 10.1186/1471-2105-14-262

**Published:** 2013-08-27

**Authors:** Yingtao Bi, Ramana V Davuluri

**Affiliations:** 1 Center for Systems and Computational Biology, Molecular and Cellular Oncogenesis Program, The Wistar Institute, 19104 Philadelphia, PA, USA

## Abstract

**Background:**

RNA-seq, a massive parallel-sequencing-based transcriptome profiling method, provides digital data in the form of aligned sequence read counts. The comparative analyses of the data require appropriate statistical methods to estimate the differential expression of transcript variants across different cell/tissue types and disease conditions.

**Results:**

We developed a novel nonparametric empirical Bayesian-based approach (NPEBseq) to model the RNA-seq data. The prior distribution of the Bayesian model is empirically estimated from the data without any parametric assumption, and hence the method is “nonparametric” in nature. Based on this model, we proposed a method for detecting differentially expressed genes across different conditions. We also extended this method to detect differential usage of exons from RNA-seq data. The evaluation of NPEBseq on both simulated and publicly available RNA-seq datasets and comparison with three popular methods showed improved results for experiments with or without biological replicates.

**Conclusions:**

NPEBseq can successfully detect differential expression between different conditions not only at gene level but also at exon level from RNA-seq datasets. In addition, NPEBSeq performs significantly better than current methods and can be applied to genome-wide RNA-seq datasets. Sample datasets and R package are available at http://bioinformatics.wistar.upenn.edu/NPEBseq.

## Background

The advent of massive parallel sequencing, popularly known as Next-Generation Sequencing (NGS), is allowing whole genomes and transcriptomes to be sequenced with extraordinary speed and accuracy, providing insights into the bewildering complexity of gene expression at both gene and isoform levels [1]. With decreasing sequencing cost per base, RNA-Seq approach has become a desirable method to get a complete view of the transcriptome and to identify differentially expressed rare transcripts and isoforms [2]. The RNA-seq assay provides sensitive and accurate digital counts for the exon regions of expressed transcripts in a given sample. The count of short sequence reads for each exon region is the sum of read counts belonging to the overlapping exon region of different transcript isoforms that are expressed in the sample. Therefore, estimating the transcript-level expression from the collection of counts of short read sequences that map to exons (or exon slices) and exon junctions is a computationally challenging problem, which has been recently attempted by us and others, in programs such as IsoformEx [3], rSeq [4], Cufflinks [5], RSEM [6], BASIS [7], and GPSeq [8]. However, none of these methods showed good agreement with qRT-PCR measurements, a gold standard in measuring differential RNA abundance between samples [3]. The statistical challenges in analyzing RNA-Seq data arise from many perspectives. While some sources of error are due to inherent problems with the technology, some are contributed at laboratory or experimental levels, leading to non-biological or technical variation across samples. Therefore, there is a critical need for investigation of other statistical methods for normalization and differential expression analysis of RNA-seq data across different conditions.

RNA-seq experiments are now frequently employed for identifying genes and alternatively spliced gene isoforms that are differentially expressed across distinct tissue/cell types and disease conditions [9]. This amounts to comparing one condition, A, with another condition, B, and producing a ranked list of differentially expressed genes according to the statistical significance of observed expression difference or fold-change between A and B [10,11]. Thus, proper normalization between samples is crucial before differential expression (DE) analysis and, to a certain degree, the two aspects are linked with each other. Normalization can be divided into within-sample normalization and between-samples normalization [12]. DE analysis is the study of the difference in absolute gene expression levels between two conditions. However, similar to microarray technology, RNA-seq is a relative abundance measure technology and does not allow for the measurement of absolute transcript abundance. This is because molecules are sampled proportionately from a large pool of cells and the initial number of cells and other technical factors are usually difficult to estimate or unknown. The standard procedure for computing the proportion of sequence reads that map to a gene relative to the total number of reads obtained in that RNA-seq experiment and for comparing those proportions across different samples can lead to high false positive rate. For example, a common method for normalization is to divide the gene-wise read counts by corresponding gene length and the total number of mapped reads to the genome. Recent reports show that the latter method, based on the total count of mapped reads, is not a robust method [13] and several alternative methods have been proposed. For example, an empirical strategy that equates the overall expression levels of genes between samples under the assumption that the majority of them are not DE was proposed recently [10,14,15]. Alternatively, the widely used quartile normalization method in the microarray field was also adapted for between-sample normalization of RNA-seq [16]. A recent review evaluated seven proposed normalization methods for the differential analysis of RNA-seq data by using a varied group of real and simulated datasets involving different species and experimental designs [13]. They concluded that the methods proposed in DESeq [17] and edgeR [18] have the most relative satisfactory behaviour compared to the others.

Similarly, several tools have been developed for DE analysis of RNA-seq data. The Poisson model has been successfully used to account for technical variations in RNA-seq data [4]. When biological replicates are available, the negative binomial distribution is commonly used to model the over-dispersion in the count data, such as DESeq and edgeR. There are also pure non-parametric methods, which do not assume any particular distribution for the data, e.g. NOISeq [11]. Approaches within a Bayesian framework for differential expression in RNA-Seq data have also been developed by many researchers, such as baySeq [19], GFOLD [20], ShrinkSeq [21] and EBSeq [22]. It is acknowledged that Bayesian approach can be used to obtain accurate and robust estimates by sharing information across all genes when sample size is small [23]. In baySeq, the genes are ranked according to the posterior probabilities of differential expression between conditions, using an empirical Bayes framework. To infer the posterior probability, the gene expression prior factors are integrated out by an approximation method [24] with mean and dispersion parameters empirically and iteratively estimated from the entire set of genes through a quasi-likelihood method. GFOLD assumes uniform prior distribution (vague prior) of gene expression level for technical replicate model. For data with biological replicates, a hierarchical model with log-normal prior for the gene expression is used to account for the biological variation. The posterior distribution of fold change is obtained through sampling. ShrinkSeq, which also takes a Bayesian perspective, is presented in a framework of generalized linear model setting, which infers the DE coefficient in the GLM directly instead of inferring the gene expression level first. ShrinksSeq explores both parametric mixture prior and non-parametric prior for the DE coefficient and extends the INLAs (integrated nested Laplace approximation) method to infer the marginal posterior distribution under non-parametric prior and shows the superior performance of non-parametric prior than parametric prior. EBSeq, similar to baySeq, ranks the genes/isoforms by posterior probability of DE, but assumes a parametric form of the prior distribution for the gene/isoform expression with parameters estimated from the data by method of moments and EM. All the aforementioned methods do not provide the close-form of posterior distribution of fold change. Because sequencing of cDNA reads is basically a sampling procedure, it is important to note that a large number of genes are unseen in a typical RNA-seq sample due to low expression or the limited depth of the experiment. For example, only approximately 0.0013% of the total number of available molecules in a RNA library are sampled in one lane of a typical Solexa/Illumina GAIIx RNA-seq experiment [25]. Further, the fact that a small number of highly expressed genes consume a significant fraction of the total sequence reads can also influence the statistical inference procedures. These limitations affect the estimation of DE or differences in relative transcript distribution between samples. For almost all currently developed RNA-seq DE methods, genes with low read counts are usually omitted from the analysis because of unreliable estimation. Another issue is that a zero read count in one condition leads to unrealistic estimation of fold change.

Here, we developed a novel method to model the RNA-seq data and detect differentially expressed genes and exons across different conditions. To mitigate the biases caused by the nature of sampling and reliably estimate the expression levels of those unseen and lowly expressed genes, we adopted a previously developed Poisson mixture model to empirically estimate the prior distributions of read counts completely from the data [26]. We propose a nonparametric, empirical Bayesian-based approach to model the RNA-seq data. We prepared five datasets, three simulated and two publicly available RNA-seq datasets, for systematically evaluating the performance of the new method. Also, the novel method is compared with the other popular methods for RNA-seq DE analysis, both using simulated and real RNA-seq datasets.

## Implementation

A few of the earlier RNA-seq assessment studies have reported highly reproducible results with little technical variation [27,28], suggesting that the inclusion of technical replicates in the experimental plan is usually not essential. Numerous RNA-Seq studies have used the Poisson model to perform testing for differential gene expression. The Poisson model assumes equality of mean and variance of read counts per gene across replicates. Therefore, pooling technical replicates together to give read counts for each biological replicate does not lead to loss of information. Thus, we first discuss one replicate per condition and then consider biological replicates.

### Model for single replicate

Let γ be the expression level of one gene under one condition and x be the number of observed reads mapped to this gene. It is well known that x follows a binomial distribution and can be approximated well by a Poisson distribution with mean λ = γdl, where 1 is the gene length and d is the normalization constant reflecting the sequencing depth. Given a prior mixing distribution G (with probability density function g(λ)) on λ, the posterior distribution of λ is $g\left(\lambda \right)\frac{\lambda^{x}e^{-\lambda d}}{x!}/h_{G}\left(x\right)$, where h_G_(x) = ∫ *λ*^*x*^/x ! e^‒ *λ*^dG(*λ*) is a G-mixture of Poisson.

A gene is expressed if, and only if, x ≥ 1. Conditioning on x ≥ 1, x follows a Q-mixture of zero-truncated Poisson h_G_(x)/(1 − h_G_(0)) or a mixture f_Q_( x ) of truncated Poisson, where

(1) $$\begin{array}{c} f_{Q}\left(x\right)=\frac{h_{G}\left(x\right)}{1-h_{G}\left(0\right)}=\int \frac{\lambda^{x}}{x!\left(e^{\lambda }-1\right)}\mathrm{dQ}\left(\lambda \right),\\ \mathrm{dQ}\left(\lambda \right)=\frac{\left(1-e^{-\lambda }\right)\mathrm{dG}\left(\lambda \right)}{\int \left(1-e^{-\eta }\right)\mathrm{dG}\left(\eta \right)}. \end{array}$$

Let n_x_ denote the number of genes with exactly x reads in the sample. The conditional nonparametric maximum likelihood estimator $\hat{Q}$ for Q is $\hat{Q}=\mathrm{argmax}\underset{x\geq 1}{\sum }n_{x}{\mathrm{logf}}_{Q}\left(x\right),$ whose calculation is discussed in [29,30] and the calculation details under the context of RNA-seq are provided in the Additional file 1. There is a one-to-one mapping between $\hat{G}$ and $\hat{Q}$ from equation (1). The posterior distribution of λ is then given by $\lambda |x\sim \hat{g}\left(\lambda \right)\frac{\lambda^{x}e^{-\lambda }}{x!}/h_{\hat{G}}\left(x\right)$. An empirical Bayes estimator for λ is

(2) $$\hat{\lambda }=E\left(\hat{\lambda }|x\right)=\frac{\left(x+1\right)h_{\hat{G}}\left(x+1\right)}{h_{\hat{G}}\left(x\right)}.$$

Let λ_A_ and λ_B_ denote the read counts that represent the true expression level of a gene and *G*_*A*_ and *G*_*B*_ denote the corresponding prior distributions, under conditions A and B, respectively. However, as mentioned previously, since NGS is like sequencing a set of sampled reads from a pool of expressed sequences of gene, the read counts that are obtained, say x_A_ and x_B_, denote the corresponding reads counts obtained in conditions A and B. The posterior distribution of log $\left(d\frac{\lambda_{A}|x_{A}}{\lambda_{B}|x_{B}}\right)$, which is log fold change of expression level of a gene, has a closed-form formula and is easy to derive, because ${\hat{G}}_{A}$ and ${\hat{G}}_{B}$ follow probability distribution of discrete form.

The normalization constant d can be inferred from some previous available methods, for example the methods proposed in DESeq or PoissonSeq [31]. This can also be calculated based on the assumption that the expected values of log-fold change of the majority of genes are zeros,

(3) $$E\left[\log\left(d\frac{\lambda_{A}|x_{A}}{\lambda_{B}|x_{B}}\right)\right]=0$$

Thus, we rank the genes by the values of $E\left[\log\left(\frac{\lambda_{A}|x_{A}}{\lambda_{B}|x_{B}}\right)\right]$ first and then estimate d by using the genes falling in the (ε, 1 − ε) quantile of all those values. In this paper, we used ε=0.25. That is, we used half of the genes to estimate d.

NPEBseq tests the hypothesis that the difference in the gene expression level between conditions A and B is above a user-defined cutoff Δ, i.e., the probability that

(4) $$\left|\log\left(d\frac{\lambda_{A}|x_{A}}{\lambda_{B}|x_{B}}\right)\right|>\Delta$$

The default value for Δ is log(2). We consider this as our own pre-defined p-value. The false discovery rate is controlled with Benjamini-Hochberg adjustment.

### Model dealing with biological replicates

RNA-seq datasets with large numbers of biological replicates are increasingly generated by many laboratories and consortia, for example, HapMap [32], ENCODE [33], and TCGA projects [34]. TCGA data consists of hundreds of RNA-seq biological replicates for each cancer condition. Dealing with the large number of biological replicate data is challenging. Recent studies have found that while the Poisson model is appropriate for technical replicates of the same RNA samples, it can be a poor fit for biological replicates. Here we propose a hierarchical Bayesian model to account for the over-dispersion in the read counts.

Let c denote the number of biological replicates for one condition and we assume that

$$\begin{array}{l} x_{\mathrm{ij}}\sim \mathrm{Poisson}\left(d_{j}e_{\mathrm{ij}}\right),\\ e_{\mathrm{ij}}\sim \mathrm{Gamma}\left(\lambda_{i},\theta \right),\mathrm{with}\;\mathrm{mean}=\lambda_{i}\;\mathrm{and}\;\mathrm{variance}=\lambda_{i}\theta ,\\ \lambda_{i}\sim G\;\mathrm{with}\;g\left(\lambda \right)\;\mathrm{denoting}\;\mathrm{the}\;\mathrm{pdf}\;\mathrm{of}\;G, \end{array}$$

where x_ij_ is the number of reads for gene i and replicate j; e_ij_ is the expression index; λ_i_ is the expression level of gene i under this condition A; θ is the scale parameter of Gamma distribution; and d_j_ is normalization constant for replicate j. The prior distribution G is inferred as before by using the sample that has the largest data depth under each condition.

Here we are interested in inferring the posterior distribution of fold change for each gene, in which the 1 will be cancelled out, so simply letting *l*=1 does not change the calculation. Let ${\vec{x}}_{i}=\left(x_{i1},x_{i2},\ldots ,x_{\mathrm{ic}}\right)$ and ${\vec{e}}_{i}=\left(e_{i1},e_{i2},\ldots ,e_{\mathrm{ic}}\right)$. Based on the aforementioned model, the joint posterior distribution of e_ij_, λ_i_ is given by

$$\begin{array}{l} \lambda_{i},{\vec{e}}_{i}|{\vec{x}}_{i}\sim g\left(\lambda_{i}\right)\overset{c}{\underset{j=1}{\prod }}\frac{1}{\Gamma \left(\lambda_{i}/\theta \right)\theta^{\frac{\lambda_{i}}{\theta }}}e_{\mathrm{ij}}{}^{\lambda_{i}/\theta -1}e^{-\frac{e_{\mathrm{ij}}}{\theta }}\frac{{\left(d_{j}e_{\mathrm{ij}}\right)}^{x_{\mathrm{ij}}}e^{-d_{j}e_{\mathrm{ij}}}}{\left(x_{\mathrm{ij}}\right)!}\\ =g\left(\lambda_{i}\right)\overset{c}{\underset{j=1}{\prod }}\frac{1}{\Gamma \left(\lambda_{i}/\theta \right)\theta^{\frac{\lambda_{i}}{\theta }}}e_{\mathrm{ij}}{}^{x_{\mathrm{ij}}+\lambda_{i}/\theta -1}e^{-e_{\mathrm{ij}}\left(\frac{1}{\theta }+d_{j}\right)}\frac{d_{j}{}^{x_{\mathrm{ij}}}}{\left(x_{\mathrm{ij}}\right)!} \end{array}$$

It is known that marginal conditional ${\vec{e}}_{i}|\left({\vec{x}}_{i},\lambda_{i}\right)$ follows Gamma distribution and can be easily integrated out (further details are given in the supplementary methods sections). Thus, the log transformed marginal posterior distribution of λ_i_ is given by

$$\begin{array}{l} \log\left(\lambda_{i}|{\vec{x}}_{i}\right)\sim \log\left(g\left(\lambda_{i}\right)\right)+\overset{c}{\underset{j=1}{\sum }}\overset{x_{\mathrm{ij}}}{\underset{k=1}{\sum }}\log\left(1+\frac{\lambda_{i}/\theta -1}{k}\right)\\ \;+\mathrm{log\theta}\underset{j}{\sum }x_{\mathrm{ij}}-\overset{c}{\underset{j=1}{\sum }}\left(x_{\mathrm{ij}}+\lambda_{i}/\theta \right)\log\left(d_{j}\theta +1\right) \end{array}$$

The p-values and FDR can be computed by equation (4).

Empirical Bayes methods have been used to estimate the degrees of over-dispersion in the data. Based on our hierarchical model, we also propose an empirical Bayes method to estimate the dispersion parameter θ. It is known that the conditional variable x_ij_|λ_i_ follows negative binomial distribution (mixture of Poisson with Gamma prior) and the expected value and variance of it are given by

$$\begin{array}{c} E\left[x_{\mathrm{ij}}|\lambda_{i}\right]=d_{j}\lambda_{i}\\ \mathrm{Var}\left[x_{\mathrm{ij}}|\lambda_{i}\right]=\lambda_{i}\left(1+{\mathrm{\theta d}}_{j}\right)d_{j}. \end{array}$$

Although the marginal distribution of x_ij_ is unclear, the expected value and variance can be computed in the following ways:

$$\begin{array}{l} E\left[x_{\mathrm{ij}}\right]=E\left[E\left[x_{\mathrm{ij}}|\lambda_{i}\right]\right]=d_{j}E\left[\lambda_{i}\right]\\ \mathrm{Var}\left[x_{\mathrm{ij}}\right]=\mathrm{Var}\left[E\left[x_{\mathrm{ij}}|\lambda_{i}\right]\right]+E\left[\mathrm{Var}\left[x_{\mathrm{ij}}|\lambda_{i}\right]\right]\\ \;=\mathrm{Var}\left[d_{j}\lambda_{i}\right]+\left(1+{\mathrm{\theta d}}_{j}\right)d_{j}E\left[\lambda_{i}\right] \end{array}$$

So we estimate θ by,

$$\frac{\mathrm{Var}\left[x_{\mathrm{ij}}\right]-d_{j}^{2}\mathrm{Var}\left[\lambda_{i}\right]}{d_{j}E\left[x_{\mathrm{ij}}\right]}-\frac{1}{d_{j}}.$$

Similar to the estimation of G, θ is also estimated by using the sample that has the largest data depth under each condition.

### Differential exon usage analysis from RNA-seq data

RNA-seq also provides information for the study of alternative splicing. DE analysis of individual transcripts is essential in many comparative studies because of isoform-level changes in gene expression between conditions [9]. Recently, two tools, Cufflink [35] and BitSeq [36], have been proposed to identify differential expression of transcripts by first estimating the expression of the transcripts. The expression or abundance estimates may contain significant correlated uncertainties that reduce the power for inference of differential expression [37]. Another tool, DEXSeq [37], proposed an exon-centric analysis to test for differential exon usage in RNA-seq data based on a generalized linear model. The input of DEXSeq is a table that contains read counts for each exon of every gene in each sample. Note that one exon may be cut into two or more parts if its boundary is not the same in all transcripts. The basic unit for counting the number of reads overlapped is called “counting bin” in this manner, similar to the definition of exon slice used in IsoformEx algorithm [3].

DEXSeq tests if each counting bin is differentially used between conditions. Inspired by this, we propose a method to detect different exon usage based on our Bayesian hierarchal model. Assuming that a gene is expressed under two different conditions, A and B, let t_Ak_ and t_Bk_ denote the expression level of counting bin k of this gene and t_Ak_ and t_Bk_ denote the observed read counts overlapping with it. The differential exon detection method involves the following steps:

1. The posterior distribution of t_Ak_|y_Ak_ and t_Bk_|y_Bk_ is derived based on our model applied to counting bin read count data.

2. Test the fitness of the distribution against the null hypothesis: the proportion of the number of reads overlapping with a counting bin to that of all the reads overlapping with the gene does not change between conditions (same as in DEXSeq).

3. Finally, define the p-value as the probability of $\left|\log\frac{t_{\mathrm{Ak}}|y_{\mathrm{Ak}}}{E\left[\lambda_{A}|x_{A}\right]}-\log\frac{t_{\mathrm{Bk}}|y_{\mathrm{Bk}}}{E\left[\lambda_{B}|x_{B}\right]}\right|>\Delta$, where Δ denotes a user-defined cutoff that represents the extent of differential expression one wishes to identify between conditions. For example, if Δ is set as log(1/3), it will test if the exon inclusion level of an exon is less than 1/3 or more than a three-fold change between conditions. Such an extreme switch of exon usage between conditions is a strong indicator of functional alterative splicing events.

### Within-condition quantile normalization

For cases without replicates, the normalization constant can be computed by equation (3). With biological replicates, d_j_ can be computed by the method proposed in DESeq or the trimmed mean of log-folder change method [10]. Here we propose within-condition quantile normalization based on the assumption that the distributions of read count data within conditions are common. The samples in the same condition are first quantile normalized relative to the one that has the largest data depth and then equation (3) is applied to do the normalization between conditions. Quantile normalization of read count data for samples coming from different conditions might not be proper due to the fact that longer genes have more reads and they might be differentially expressed between conditions, which can put the gene expression level in a different scale within one sample. We compared this within-condition quantile normalization to the method proposed in DESeq for the simulated data and no significant difference was present (results are not shown here). For rest of this paper, we adopt the within-condition quantile normalization procedure.

### Datasets for simulation

We generated three simulation datasets to evaluate the proposed method for identifying differentially expressed genes. Dataset1 is generated with biological replicates by assuming different priors across conditions, which means *G*_*A*_ and G_B_ follow different statistical distributions. Dataset2 consists of no replicates and is generated by following the simulation scheme adopted by baySeq and edgeR. Dataset3 is generated by the same scheme as dataset2 but with biological replicates.

## Results

### NPEBseq method

**Figure 1:**
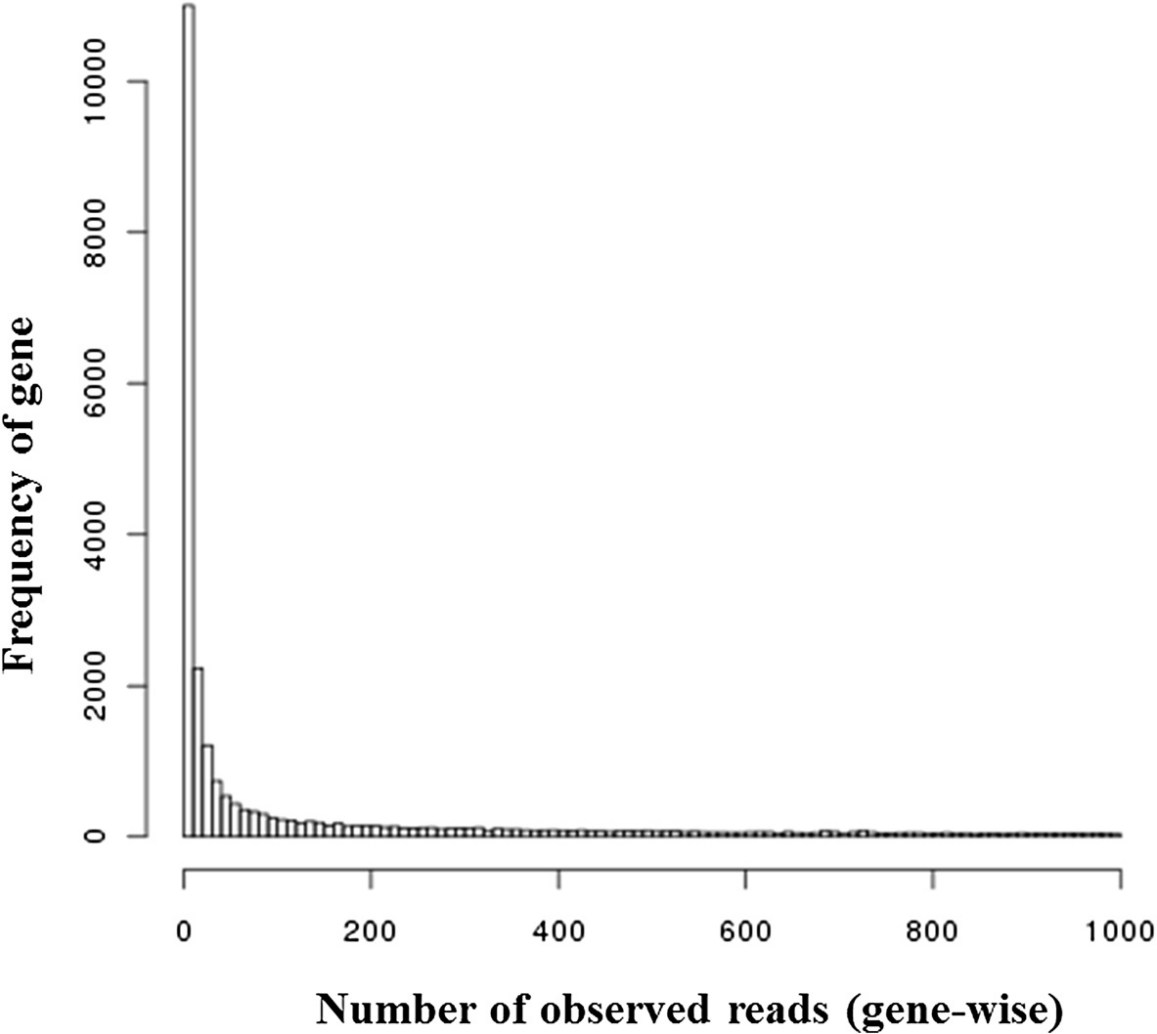
**Distribution of number of observed reads per gene for genes with read count less than 1000.** The number of genes in a RNA-seq dataset is shown in relation to number of mapped reads per gene. X-axis: number of observed reads per gene; Y-axis: frequency of genes.

NPEBSeq is a nonparametric empirical Bayesian-based approach to model the RNA-seq data. The expression level of genes with low read counts is estimated by borrowing information from the gene expression in the whole sample. The non-parametric form of the prior distribution avoids any unrealistic assumption. The parametric assumption for the prior distribution is usually not fulfilled for the RNA-seq read count data. The fact that there are many genes expressed at low levels in one sample is illustrated in Figure 1, which is generated based on one sample from Marioni’s RNA-seq dataset [27]. This plot clearly shows that a large proportion of genes in a sample are expressed at low levels. These genes could have a high impact on the performance of statistical methods to identify differentially expressed genes. The fact that there are large numbers of genes/transcripts with low read counts and a small number of genes with a significantly high number of reads make any parametric assumption for the prior distribution unrealistic.

### Performance of NBESeq on simulated datasets

To evaluate the proposed method for identifying differentially expressed genes, we first conducted a simulation study.

#### Simulation 1 - Simulation with different priors between conditions (dataset1)

To generate data similar to those produced by real RNA-seq experiments, we first applied the empirical Bayes method on publicly available RNA-seq datasets, which were generated to compare liver and kidney transcriptomes [27]. The prior distributions of kidney and liver samples were first estimated and then the data was normalized based on the expected values. The corresponding dispersion parameter θ for each condition was also estimated.

Dataset1 consist of 20 independent simulations with seven samples each for two conditions. The library size of each sample is uniformly sampled from 300,000 to 900,000. Each sample was generated by a mixture of negative binomial model with both the prior distributions and dispersion parameters estimated from Marioni’s data. Each sample consists of 10,000 genes for computational efficiency.

We performed a comparative analysis of our method with four popular methods, DESeq, edgeR, baySeq and NOISeq, which are available as part of Bioconductor packages at http://bioconductor.org[38]. The edgeR implements two ways to estimate the dispersion parameter in its model, common dispersion and tag-wise dispersion. Both of them are studied here. baySeq provides two choices of model (Poisson and negative binomial). We adopted the negative binomial model for dataset1. Both DESeq and edgeR provide p-values for ranking the genes. baySeq provides log posterior likelihood ratio for ranking the differential expression of genes. In the case of NPEBseq, we rank the genes by p-values as defined in equation (4). The purpose of this simulation is to compare the ability of these methods to rank the genes in order of differential expression. The true ranking order of the genes is based on the fold change of differential expression values between the two conditions.

**Figure 2:**
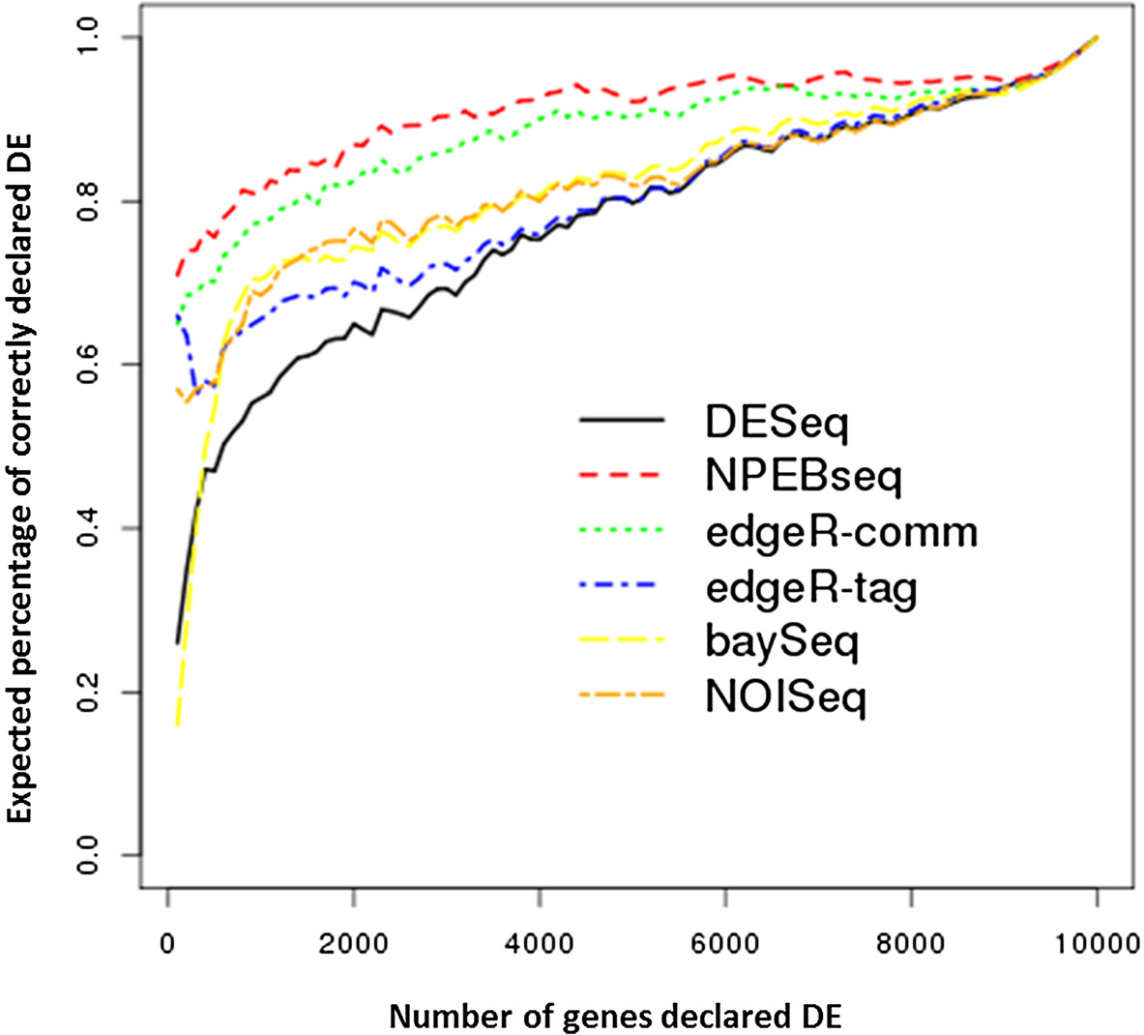
**Simulation results of comparing the performance of DESeq, edgeR and NPEBseq on dataset1.** The x axis denotes τ and y axis denotes p_τ_.

We used the following criteria to compare the performance of different methods. Given a cutoff point τ (e.g. the number of genes declared significantly expressed), the efficiency of a statistical method is measured by p_τ_, the expected percentage of the true first τ DE genes being correctly declared as the first τ DE genes. The average of estimated p_τ_ is calculated from the 20 replicates. The simulation results for dataset1 are shown in Figure 2. The proposed NPEBseq method outperformed other methods.

#### Simulation 2 - simulation with the same priors between conditions (dataset2 and dataset3)

A simulation scheme similar to the one suggested by Robinson and Smyth [39] is applied here to generate dataset2 and dataset3. The library size of each sample was uniformly sampled from 300,000 to 900,000. The prior distribution of λ was assumed to be common between the two conditions and estimated from the liver RNA-seq data of Marioni.

**Figure 3:**
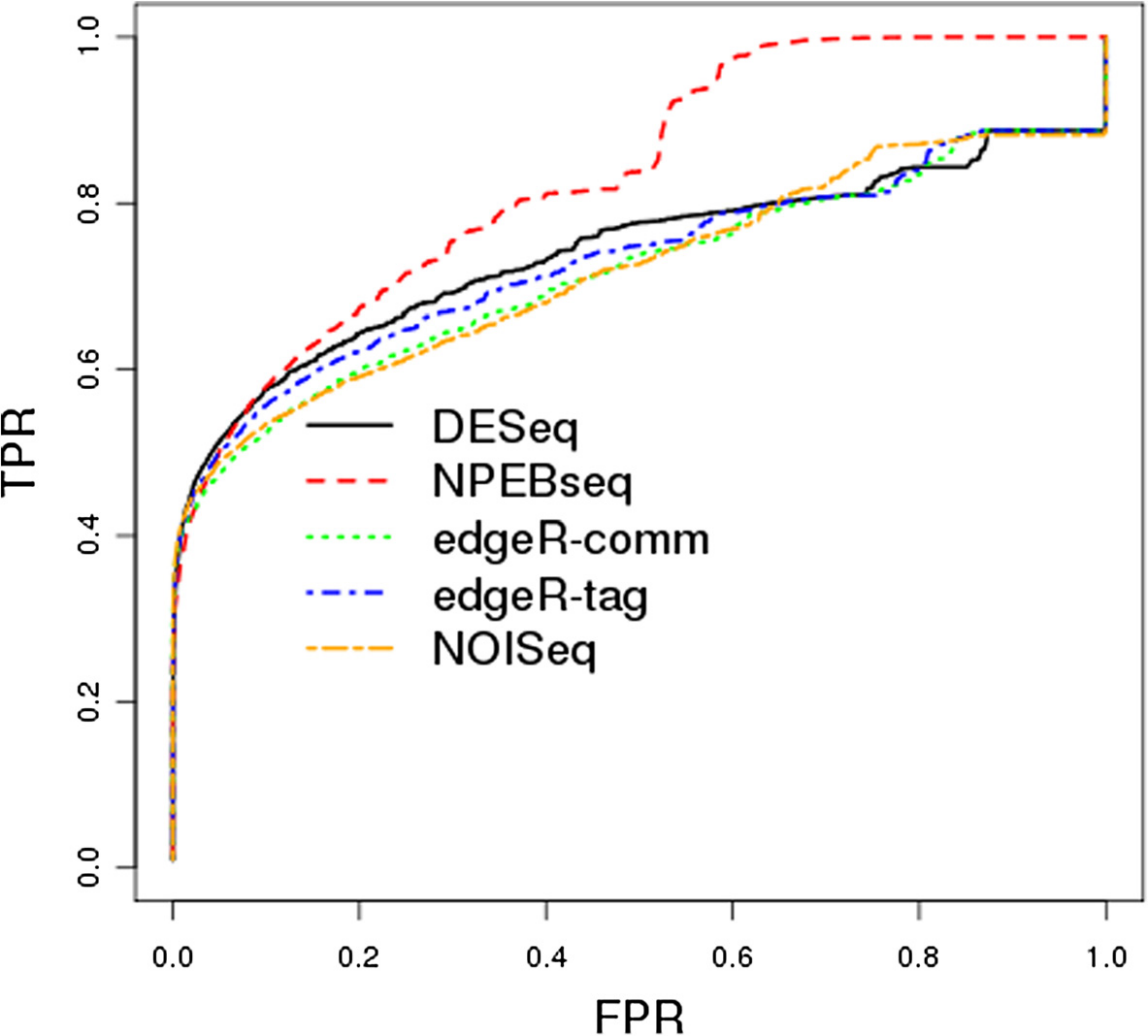
**ROC curves based on simulated dataset2.** The programs evaluated are: DESeq, edgeR, NPEBseq and NOISeq. The method baySeq is not shown due to its poor performance on dataset without replicates.

**Figure 4:**
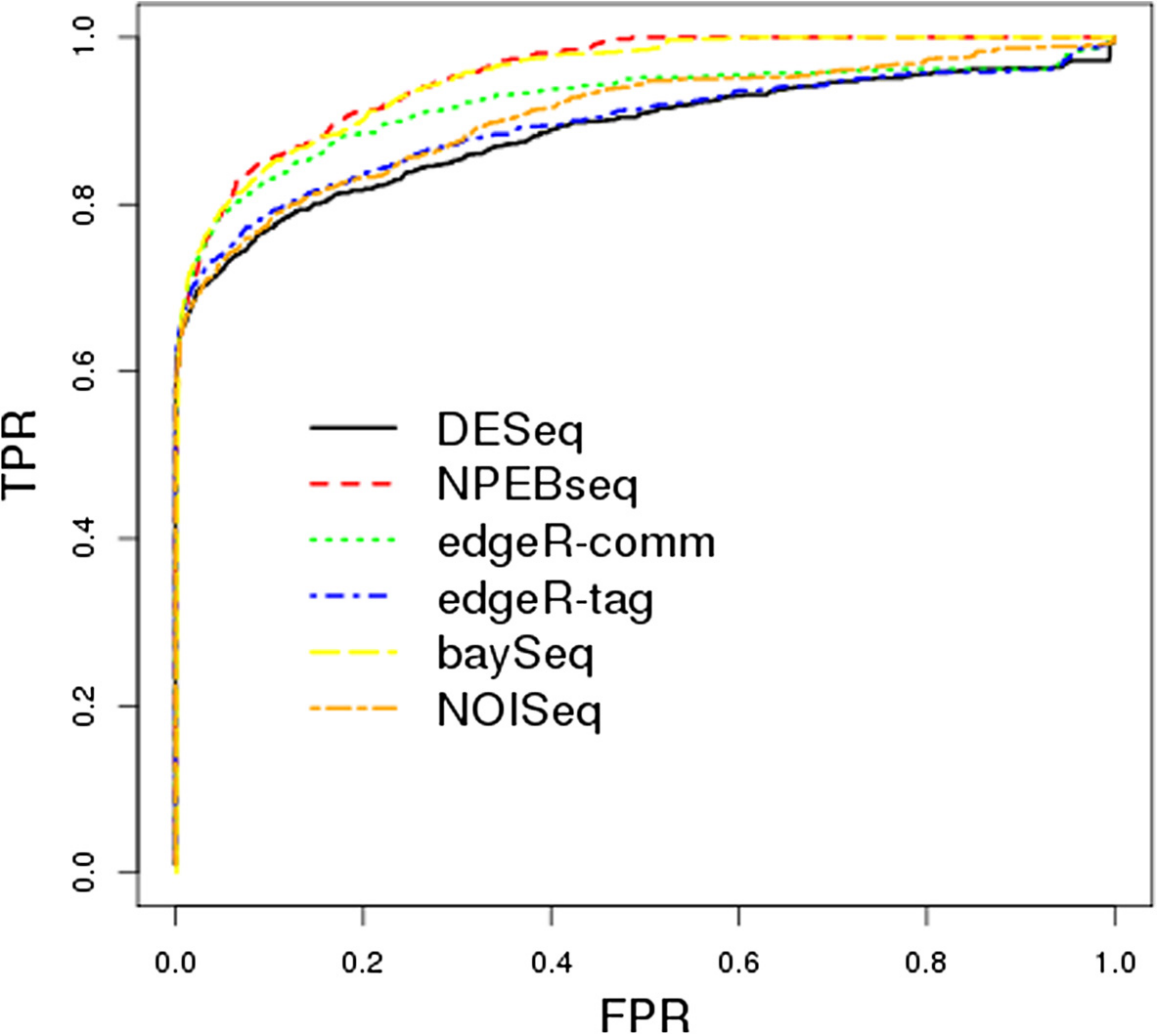
**ROC curves based on simulated dataset3.** The programs evaluated are: DESeq, edgeR, baySeq, NPEBseq and NOISeq.

Dataset2 was generated by Poisson distribution and dataset3 by negative binomial distribution, with the dispersion parameter estimated from the liver data. The simulated data consists of 10,000 genes, and one-tenth of those genes were set to be differentially expressed (between condition A and condition B) with λ_A_ = bλ_B_. In order to produce both over- and under-expression in our simulated data, 500 randomly selected genes were set to have b=4 and the remaining 500 genes were set to have b=1/4. Both dataset2 and dataset3 consist of 20 independent simulations. Dataset2 was generated without replicates. Similar to dataset1 seven samples per condition per simulation were generated for dataset3. The full ROC curves for dataset2 and dataset3 are shown in Figures 3 and 4, respectively. Based upon examination of these curves, the proposed NPEBseq method appears to perform better than the other methods. The partial ROC curves with false positive rate less than 0.2 are shown in Additional file 2: Figure S1 and Figure S2, which indicate that NPEBSeq performs as well as the other methods. To clearly show that NPEBSeq can robustly estimate fold change of genes with low read counts, the estimated fold change of 10 genes from one sample of dataset2 by NPEBseq along with DESeq and edgeR are shown in Table 1. For the cases with zero read count under one condition, DESeq always gives infinite estimation of fold change.

**Table 1:** Estimated fold change of 10 genes from one sample of simulated dataset2 using NPEBSeq, DESeq and edgeR

gene_ID	9995	9996	9511	9032	9045	9030	9082	3	1
Poisson mean under condition A	0.7741	1.4868	11.5416	424.1334	5.2419	5.2419	112.1307	0.7741	86.6691
Poisson mean under condition B	3.0318	5.8233	45.2038	103.8228	1.2832	1.2832	27.4483	0.758	84.8622
TRUE fold change	4	4	4	4	4	4	4	1	1
observed #reads under condition A	0	0	2	365	34	13	89	0	80
observed #reads under condition B	4	2	37	111	0	1	83	4	72
estimated fc by NPEBSeq	2.5813	1.8077	13.2633	3.3515	18.9702	5.8962	1.1168	2.5813	1.0156
estimated fc by DESeq	inf	inf	18.6932	3.2543	inf	12.8656	1.0612	inf	1.0996
estimated fc by edgeR	60.4244	24.121	58.9655	5.3961	1192.127	29.3642	1.0736	60.4244	1.1299

### Real RNA-seq data analysis

To further evaluate our method, we tested it on two published RNA-seq datasets.

#### Real RNA-seq data 1–Comparison based on one MAQC dataset

We first applied NPEBSeq on the MicroArray quality control (MAQC) dataset [40,41] and compared with DESeq, baySeq, and edgeR. MAQC datasets contain gene expression data from multiple platforms and are extensively used in evaluating different data processing methods. We downloaded the MAQC2 Illumina RNA-seq data from http://www.ncbi.nlm.nih.gov/sra, which contains seven technical replicates of brain reference RNA samples and seven technical replicates of UHR RNA samples. Tophat [35] was used for tag alignment and counts for each gene were computed by means of HTSeq Python package (http://www-huber.embl.de/users/anders/HTSeq/), using the annotation of the Ensembl genes and only reads that mapped to exons.

As part of the original MAQC project, around 1,000 genes were also chosen to be assayed by Taqman qRT-PCR. Those qRT-PCR data were obtained from GEO database, which contains four technical replicates for each of the two samples. The qRT-PCR data were used as a gold standard to benchmark the gene expression values by RNA-seq. We analysed the qRT-PCR data using the comparative C_t_ methods [42]. Finally, 407 genes were defined as DE and 119 genes were defined as non-DE. Given the fact that not all the genes were assayed by qRT-PCR, we followed the same procedure that was applied in [15] to define the true positive and false positive rates. Given a “DE” or “non-DE” call from qRT-PCR, define a true positive (TP) as the event that the test of interest calls a gene DE that qRT-PCR called DE. A false positive (FP) event occurs when the test calls a gene DE that qRT-PCR called non-DE. The true positive rate (TPR) is defined as

$$\frac{\left(\#\mathrm{TP}\;\mathrm{and}\;\mathrm{qRT}-\mathrm{PCR}\;\mathrm{is}\;\mathrm{DE}\right)/\left(\mathrm{total}\#\mathrm{genes}\right)}{\#\mathrm{qRT}-\mathrm{PCR}\;\mathrm{is}\;\mathrm{DE}/\left(\mathrm{total}\#\mathrm{genes}\right)}$$

and the false positive rate (FPR) is defined as

$$\frac{\left(\#\mathrm{FP}\;\text{and}\;\text{qRT}-\text{PCR}\;\mathrm{is}\;\text{non}-\mathrm{DE}\right)/\left(\text{total}\#\text{genes}\right)}{\#\text{qRT}-\text{PCR}\;\mathrm{is}\;\text{non}-\mathrm{DE}/\left(\text{total}\#\text{genes}\right)}$$

Note that these are not the standard definitions of TPR and FPR.

qRT-PCR data were annotated by RefSeq. The BioMart R package [43] was used to convert the RefSeq genes IDs for qRT-PCR to Ensembl genes ids.

**Figure 5:**
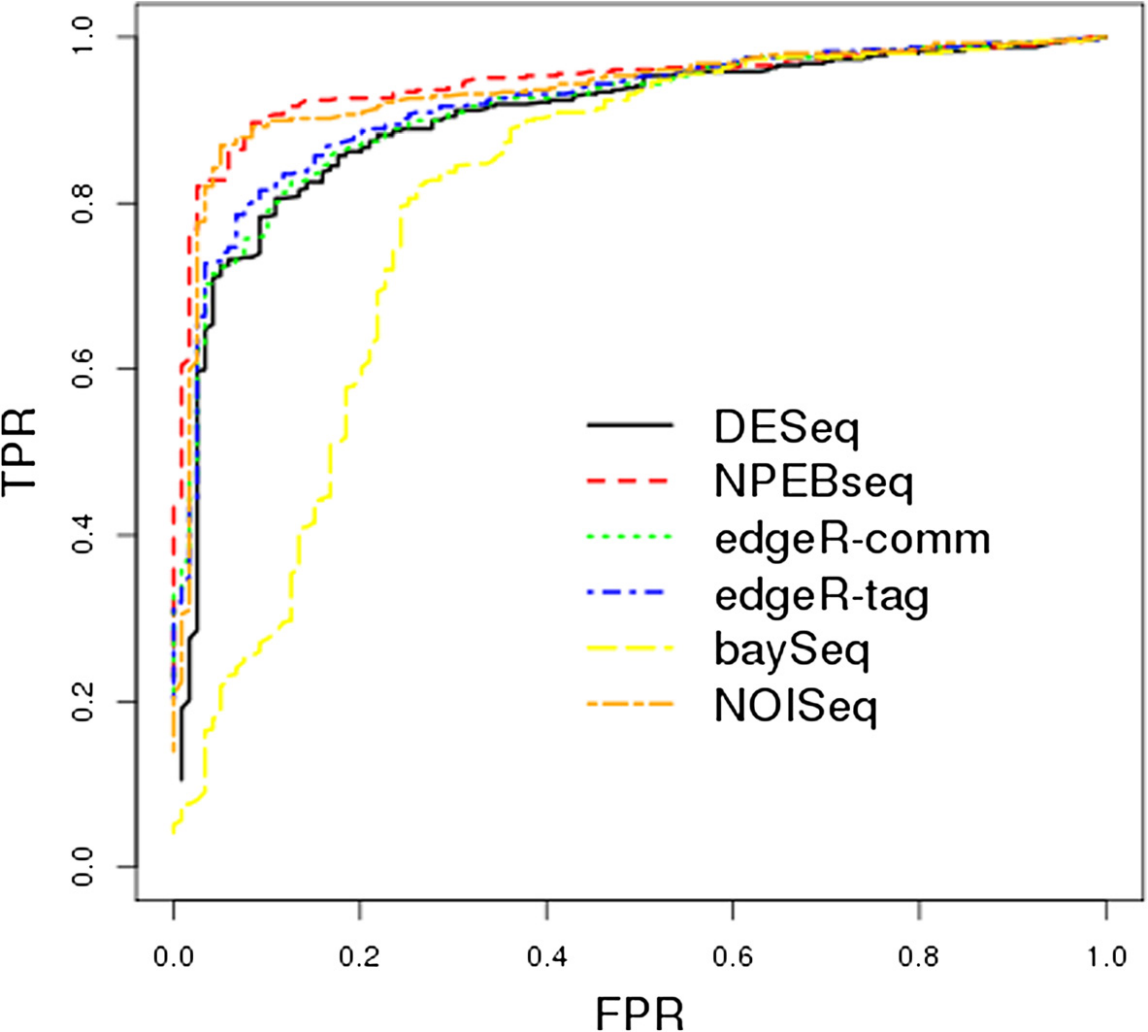
**ROC curves based on MAQC2 real RNA-seq data: Comparison of the performance of DESeq, edgeR, baySeq, NPEBseq and NOISeq methods.** We declared non-DE if its RT-qPCR absolute log-ratio was less than 0.2 and DE if its absolute log-ratio was greater than 2.0.

The ROC curves from all the compared methods are shown in Figure 5. Clearly, our proposed method has the best performance in terms of sensitivity and specificity.

#### Real RNA-seq data 2–Detecting differential usage of exons from RNA-seq data

We also analysed the data by Brook et al. [44], where the effect of the RNAi knockdown of “pasilla” was studied by RNA-seq in the Drosophila melanogaster cell line. The data was downloaded as part of DEXSeq package. The data consists of four control samples and three knockdown samples. The analysis at gene level by NPEBseq reported 107 differentially expressed genes, with nominal FDR control at 0.1 for the comparison of control and knockdown. To access the specificity of the NPEBseq method we performed in-condition comparison by making use of the fact that there are four biological replicates in the control group. We applied NPEBseq for the comparison of two control samples versus the other two. NPEBseq reported zero differentially expressed genes with FDR control at 0.1, which indicates that NPEBseq has a very high specificity.

**Figure 6:**
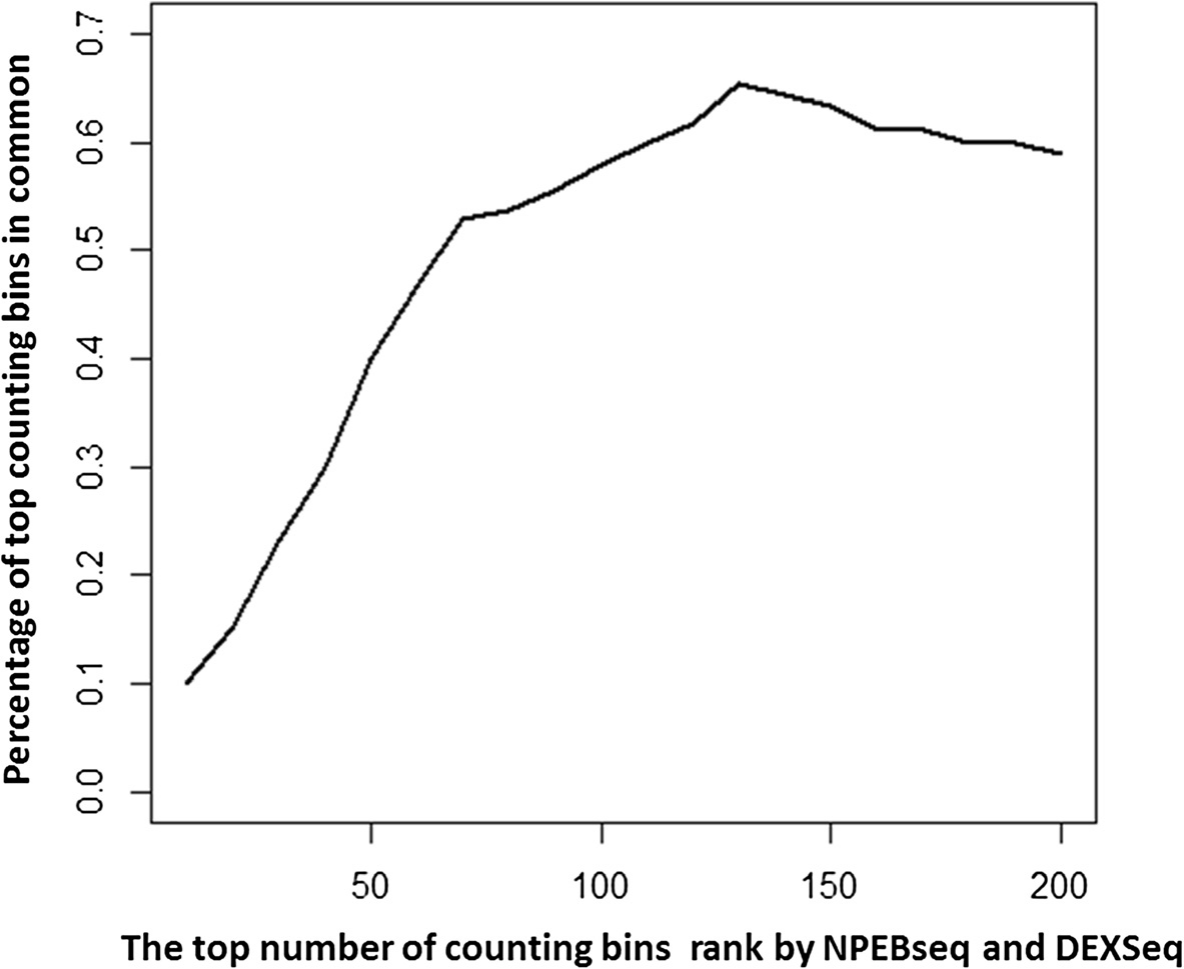
**Percentage of DE exons that are common in the two ranked lists of exons generated by NPEBseq and DEXSeq programs.** While x-axis denotes the number of declared DE exons and the y-axis denotes the percentage of common calls between both the programs.

We then analysed Brook’s data at exon level. NPEBseq found differential exon usage for 2,370 counting bins at FDR 0.01 for between-condition comparison and 225 counting bins for within-condition comparison. We also applied the newest version of DEXSeq on the exon data, which reported 120 counting bins as DE at FDR 0.1. We checked whether NPEBseq and DEXSeq could achieve comparable results by computing the percentage of DE called exons that are common in the two ranked lists of exons generated by both programs. The results are shown in Figure 6. For example, we found that 74 counting bins (exons) were common among the top 120 DE counting bins called by each approach. And, further examination revealed that among the top 120 DE counting bins identified by NPEBseq, 12 were defined as “untestable” by the DEXSeq method due to low read counts in those counting bins. Since the p-value defined in NPEBseq is different from the regular p-value, we didn’t expect these two approaches to report similar number of DE exons at the same FDR level.

## Discussion

In this paper we developed a novel empirical Bayesian-based approach to model the RNA-seq data. This method has been widely used in ecology to estimate species diversity [26]. The nonparametric form of the prior distribution of the Bayesian model is empirically estimated from the data. The expression level of genes with low read counts are estimated by borrowing information from the gene expression in the whole sample. For data with biological replicates, we developed a hierarchal Bayesian model to account for the over-dispersion and proposed an empirical Bayesian method to estimate the dispersion parameter. We also extended the model to detect differential usage of exons from RNA-seq datasets. The closed-form formula of the posterior distribution makes the computation of any statistics very efficient. At the final step, we evaluated the performance of this method in detecting the differentially expressed genes by conducting simulation and real RNA-seq data analysis.

There are many challenges still present in the processing and analyses of RNA-seq data. For example, it has been empirically observed that quantification of expression depends on the length of the biological features under study (genes, transcripts, or exons), as longer features tend to have more significant statistics than shorter ones [45]. Also, it was recently shown that there exists a sample-specific guanine-cytosine content (GC-content) effect and the studies proposed normalization methods by GC-strata to remove such effects [46,47]. Incorporating those factors into our model could further improve the performance.

Delineating the gene expression at an alternative transcript-level from RNA-seq data is still a very challenging problem. Our recently published IsoformEx method [3], based on non-negative least square, is aimed to estimate transcript abundance. In future enhancements to the proposed method we will integrate NPEBseq with IsoformEx to identify DE at isoform-level.

## Conclusions

NPEBseq can be applied to not only detect differential gene expressions from the RNA-seq dataset with technical and biological replicates for both studied conditions, but also to detect differential usage of exons. It is robust, since it requires no limited assumptions to be made about the prior distribution of the data. NPEBseq also provides the closed form of posterior distribution of the fold change, which is useful for further analysis.

## Availability and requirements

**Project name:** NPEBseq

**Project home page:**http://bioinformatics.wistar.upenn.edu/NPEBseq

**Operating system and R version:** The R package is platform independent and is compatible with all the versions of R same as or higher than 2.15.1.

**Other requirements:** No.

**License:** GPL (≥ 2)

**Any restrictions to use:** It is available for free download.

## Competing interests

Both authors declare that they have no competing interests.

## Authors’ contributions

YB and RVD conceived the initial approach. YB designed and implemented the methods and performed the analyses. YB and RVD wrote the manuscript. All authors read and approved the final manuscript.

## Supplementary Material

Additional file 1 **This file describes the procedure to derive the marginal posterior distribution of λ**_**i **_**and the procedure to infer the prior distribution of our proposed model.** Click here for file

Additional file 2: Figure S1 Partial ROC curves based on simulated dataset2. **Figure S2** Partial ROC curves based on simulated dataset3. Click here for file
