# Supplementary material for: NPEBseq: nonparametric empirical bayesian-based procedure for differential expression analysis of RNA-seq data
Source: BMC Bioinformatics. 2013 Aug 27;14:262. doi: 10.1186/1471-2105-14-262 (PMC3765716; doi:10.1186/1471-2105-14-262)
Supplement: Additional file 1 — This file describes the procedure to derive the marginal posterior distribution of λi and the procedure to infer the prior distribution of our proposed model. [file 1471-2105-14-262-S1.docx]

**Supplementary text**

1. **The procedure to derive the marginal posterior distribution of** $\boldsymbol{\lambda}_{\boldsymbol{i}}$**.**

For a given gene $i$, the joint posterior distribution of $e_{ij}$, $\lambda_{i}$ is

$$\lambda_{i},\vec{e_{i}}|\vec{x_{i}}\sim g\left( \lambda_{i} \right)\prod_{j=1}^{c} \frac{1}{\Gamma\left( {\lambda_{i}}/\theta\right)\theta^{\frac{\lambda_{i}}{\theta}}}{e_{ij}}^{{\lambda_{i}}/\theta-1}e^{-\frac{e_{ij}}{\theta}}\frac{{{(d_{j}e}_{ij})}^{x_{ij}}e^{-{d_{j}e}_{ij}}}{\left( x_{ij} \right)!}$$

$$=g\left( \lambda_{i} \right)\prod_{j=1}^{c} \frac{1}{\Gamma\left( {\lambda_{i}}/\theta\right)\theta^{\frac{\lambda_{i}}{\theta}}}{e_{ij}}^{x_{ij}+{\lambda_{i}}/\theta-1}e^{-e_{ij}(\frac{1}{\theta}+d_{j})}\frac{{d_{j}}^{x_{ij}}}{\left( x_{ij} \right)!}$$

Base on the fact that marginal conditional $\vec{e_{i}}|(\vec{x_{i}},\lambda_{i})$ follows Gamma distribution, the terms with $e_{ij}$ can be easily integrated out by

$$\int{e_{ij}}^{x_{ij}+{\lambda_{i}}/\theta-1}e^{-e_{ij}(\frac{1}{\theta}+d_{j})}=\Gamma(x_{ij}+{\lambda_{i}}/\theta){(\frac{\theta}{d_{j}\theta+1})}^{x_{ij}+{\lambda_{i}}/\theta}$$

Thus marginal posterior distribution of $\lambda_{i}$ is

$$\lambda_{i}|\vec{x_{i}}\sim g\left( \lambda_{i} \right)\prod_{j=1}^{c} \frac{1}{\Gamma\left( {\lambda_{i}}/\theta\right)\theta^{\frac{\lambda_{i}}{\theta}}}\Gamma(x_{ij}+{\lambda_{i}}/\theta){(\frac{\theta}{d_{j}\theta+1})}^{x_{ij}+{\lambda_{i}}/\theta}\frac{1}{\left( x_{ij} \right)!}$$

$$\sim g\left( \lambda_{i} \right)\prod_{j=1}^{c} \frac{\Gamma(x_{ij}+{\lambda_{i}}/\theta)}{\Gamma\left( {\lambda_{i}}/\theta\right)}\frac{\theta^{x_{ij}}}{{(d_{j}\theta+1)}^{x_{ij}+{\lambda_{i}}/\theta}}\frac{1}{\left( x_{ij} \right)!}$$

We also have the following property for gamma function:

$$\frac{\Gamma\left( x_{ij}+{\lambda_{i}}/\theta\right)}{\Gamma\left( {\lambda_{i}}/\theta\right)}\frac{1}{\left( x_{ij} \right)!}=\left( x_{ij}+{\lambda_{i}}/\theta-1 \right)\ldots.{\lambda_{i}}/\theta\frac{1}{\left( x_{ij} \right)!}$$

$$=\left( 1+\frac{{\lambda_{i}}/\theta-1}{x_{ij}} \right)\ldots.(1+\frac{{\lambda_{i}}/\theta}{1})$$

$$=\prod_{k=1}^{x_{ij}} (1+\frac{{\lambda_{i}}/\theta-1}{k})$$

Thus the log transformed marginal posterior distribution of $\lambda_{i}$ is given by

$${log(\lambda}_{i}|\vec{x_{i}})\sim\log\left( g\left( \lambda_{i} \right) \right)+\sum_{j=1}^{c} \sum_{k=1}^{x_{ij}} log(1+\frac{{\lambda_{i}}/\theta-1}{k})+log\theta\sum_{j} x_{ij}-\sum_{j=1}^{c} (x_{ij}+{\lambda_{i}}/\theta)log(d_{j}\theta+1)$$

1. **The procedure to infer the nonparametric prior distribution, G.**

Considering a cDNA library comprising M expressed genes. A RNA-seq experiment is conducted and one sample is taken. Let $x_{i}$ be the number of observed reads mapped to gene i with $i=1,2,\ldots,N$, where N is total number of observed genes. It is important to note that N is a known number and M is unknown. Let $n_{x}$ denote the number of genes with exactly x reads in the sample. Because gene i is unseen when $x_{i}=0$, $n_{0}$ denotes the number of genes unseen in the sample and we have the following equation, $N=\sum_{x=1}^{\infty} n_{x}=M-n_{0}$.

Considering a gene i, it is well known that $x_{i}$ follows a binomial distribution and can be approximated well by a Poisson distribution with mean $\lambda_{i}$. Assuming a prior mixing distribution $G$ on $\lambda_{i}$, the $x_{i}$’s arise as a sample from a Poisson mixture and all $x_{i}$’s are iid observations from $h_{G}\left( x \right)=\int{e^{-\lambda}\lambda^{x}}/{(x!)dG(\lambda)}$. Here we assume G is in an unknown nonparametric form (discrete distribution) and we are interested in inferring it from the data.

The full likelihood of the number of genes M and the mixing distribution G is

$L\left( G,M \right)=\frac{M!}{\left( M-N \right)!\prod_{x=1}^{\infty} n_{x}!}h_{G}^{M-N}(0)\prod_{x=1}^{\infty} h_{G}^{n_{x}}(x)$,

which is a multinomial density function. It is known that this likelihood function can be factored into two parts,

$$L\left( G,M \right)=\left( \begin{matrix} M \\ N \end{matrix} \right)h_{G}^{M-N}\left( 0 \right)\left[ 1-h_{G}\left( 0 \right) \right]^{N}\times\frac{N!}{\prod_{x=1}^{\infty} n_{x}!}\prod_{x=1}^{\infty} \left( \frac{h_{G}\left( x \right)}{1-h_{G}\left( 0 \right)} \right)^{n_{x}}=L_{1}\left( G,M \right)\times L_{2}\left( G \right).$$

Here the likelihood $L_{1}\left( G,M \right)$ is from the binomial marginal distribution of N, which depends on both G and M. The conditional distribution of $x_{i}$ ($i=1,2,\ldots,N$) given M generates $L_{2}\left( G \right)$, which depends on G alone. Mao and Lindsay [[1](#_ENREF_1)] identified that the conditional log-likelihood can be reparameterized into a Q-mixture of 0-truncated Poisson densities as $l_{2}\left( Q \right)=\sum_{x=1}^{\infty} n_{x}\log f_{Q}\left( x \right),$ where,$f_{Q}\left( x \right)=\frac{h_{G}\left( x \right)}{1-h_{G}(0)}=\int\frac{\lambda^{x}}{x!\left( e^{\lambda}-1 \right)}\mathrm{dQ}\left( \lambda\right)$ and $\mathrm{dQ}\left( \lambda\right)=\frac{\left( 1-e^{-\lambda} \right)dG(\lambda)}{\int\left( 1-e^{-\eta} \right)dG(\eta)}.$ Thus Q-G is a one-to-one transformation. Q also has a discrete form since the discrete form of G. The advantage of the form $l_{2}\left( Q \right)$ is that it is standard non-parametric mixture likelihood [[2](#_ENREF_2)] of iid observation from a mixture of 0-truncated Poisson variable. The properties of the NPMLE (non-parametric MLE) $\hat{Q}$ were detailed in [[3](#_ENREF_3)]. A numerical algorithm to infer Q was proposed in [[4](#_ENREF_4)] through a combination of EM (expectation-maximization) and VEM (vertex-exchange method) algorithms. Given an initial estimate of Q, the EM algorithm is used to increase the likelihood and the VEM is used to update the number of support points in Q. Iterating between the EM stages and VEM stages leads to a fast, reliable hybrid procedure [[5](#_ENREF_5)].

**References**

1. Mao CX, Lindsay BG: **Tests and diagnostics for heterogeneity in the species problem**. *Comput Stat Data An* 2003, **41**(3-4):389-398.

2. Lindsay BG: **Mixture models : theory, geometry, and applications**. Hayward, Calif.Alexandria, Va.: Institute of Mathematical Statistics ;American Statistical Association; 1995.

3. Mao CX: **Predicting the conditional probability of discovering a new class**. *Journal of the American Statistical Association* 2004, **99**(468):1108-1118.

4. Mao CX: **Inference on the number of species through geometric lower bounds**. *Journal of the American Statistical Association* 2006, **101**(476):1663-1670.

5. Bohning D: **A Review of Reliable Maximum-Likelihood Algorithms for Semiparametric Mixture-Models**. *J Stat Plan Infer* 1995, **47**(1-2):5-28.
