# Supplementary material for: NPEBseq: nonparametric empirical bayesian-based procedure for differential expression analysis of RNA-seq data
Source: BMC Bioinformatics. 2013 Aug 27;14:262. doi: 10.1186/1471-2105-14-262 (PMC3765716; doi:10.1186/1471-2105-14-262)
Supplement: Additional file 2: Figure S1 — Partial ROC curves based on simulated dataset2. Figure S2 Partial ROC curves based on simulated dataset3. [file 1471-2105-14-262-S2.docx]

**Supplementary Figure:**

**Figure S1 – Partial ROC curves based on simulated dataset2**

The programs evaluated are: DESeq, edgeR, NPEBseq and NOISeq.

**Figure S2 – Partial ROC curves based on simulated dataset3**

The programs evaluated are: DESeq, edgeR, baySeq, NPEBseq and NOISeq.


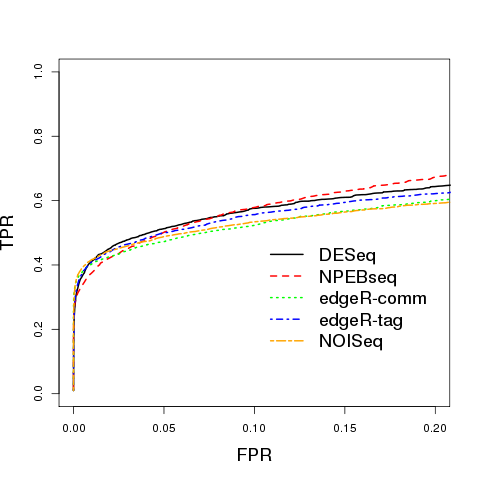


**Figure S1**


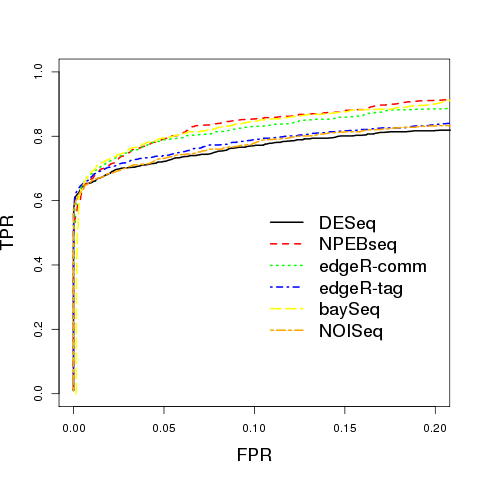


**Figure S2**
